# Relapsing/remitting type 1 diabetes

**DOI:** 10.1007/s00125-017-4403-3

**Published:** 2017-08-23

**Authors:** Kayleigh M. van Megen, Matthew P. Spindler, Fleur M. Keij, Ineke Bosch, Fleur Sprangers, Annet van Royen-Kerkhof, Tatjana Nikolic, Bart O. Roep

**Affiliations:** 1 Department of Diabetes Immunology, Diabetes & Metabolism Research Institute, Beckman Research Institute at the City of Hope, 1500 E Duarte Rd, Duarte, CA 91010 USA; 2 Department of Immunohaematology and Blood Transfusion, Leiden University Medical Center, Leiden, the Netherlands; 3 DeKinderkliniek, Children’s Hospital, Almere, the Netherlands; 4 Flevo Hospital, Almere, the Netherlands; 5 Department of Pediatric Immunology and Rheumatology, Wilhelmina Children’s Hospital, University Medical Center Utrecht, Utrecht, the Netherlands

**Keywords:** Autoimmune disease, Immune regulation, Immunotherapy, Type 1 diabetes

## Abstract

**Aims/hypothesis:**

Type 1 diabetes is believed to be an autoimmune disease associated with irreversible loss of insulin secretory function that follows a chronic progressive course. However, it has been speculated that relapsing/remitting disease progression may occur in type 1 diabetes.

**Methods:**

We report the case of an 18-year-old girl with Graves’ disease, chronic inflammatory demyelinating polyneuropathy (CIDP) and multiple islet autoantibodies, presenting with relapsing/remitting hyperglycaemia. Peripheral blood mononuclear cells were analysed for islet autoimmunity.

**Results:**

There were two instances of hyperglycaemia relapse during CIDP flare-ups that required insulin therapy and remitted after i.v. immunoglobulin (IVIG) therapy improving neurological symptoms. A diagnosis of type 1 diabetes was assigned on the basis of insulin need, HbA_1c_ and islet autoantibodies. Insulin requirements disappeared following IVIG treatment and peaked during CIDP flare-ups. Pro- and anti-inflammatory cytokine responses were noted against islet autoantigens.

**Conclusions/interpretation:**

We provide clinical evidence of relapsing/remitting type 1 diabetes associated with IVIG treatment and the regulation of islet autoimmunity. Despite sufficient residual beta cell mass, individuals can experience episodes of impaired glycaemia control. This disconnect between beta cell mass and function highlighted by our case may have implications for the use of beta cell function as the primary endpoint for immune intervention trials aiming to protect beta cell mass rather than function. Immune modulation may restore beta cell function and glycaemic control.

## Introduction

Studies on cohorts of people with recent onset type 1 diabetes suggest steadily declining pancreatic beta cell function over time. However, these data may not necessarily reflect the dynamics of disease progression in individuals [1, 2]. Here we present an individual with relapsing/remitting type 1 diabetes with regulated islet autoimmunity.

## Methods

In February 2013, a 15-year-old girl presented with muscle weakness, numbness in the arms and legs, and areflexia. Blood tests revealed elevated blood sedimentation rate, thrombocytosis and initially normal serum glucose. Elevated protein levels were observed in the spinal fluid. Collectively indicating a diagnosis of chronic inflammatory demyelinating polyneuropathy (CIDP). She was treated with i.v. immunoglobulin’s (IVIG) for 5 days with good results. CIDP is characterised as a relapsing disorder which develops over a period of several weeks. The classical clinical pattern of the disease is described by the American Academy of Neurology and consists of involvement of proximal and distal limbs and of both motor and sensory fibres [3].

Proliferating peripheral blood mononuclear cells (PBMCs) were measured by ^3^H-labelled thymidine uptake using a previously published method [4]. PBMCs were pulsed with antigens relevant to type 1 diabetes (preproinsulin [PPI], islet antigen-2 [IA-2] or glutamic acid decarboxylase 65 [GAD65]). Tetanus toxoid was used as a positive control. Human serum albumin was used as a negative control, to which the proliferation data were normalised by subtracting the negative control values from the diabetes-relevant antigen values. Recombinant PPI, IA-2 and GAD65 were purified from *E. coli* and tested negatively for endotoxin contamination. Supernatant fractions from proliferation cultures were collected and cytokine production measured using a nine-plex Luminex kit (Biorad, Veenendaal, the Netherlands). Autoantibody titres were measured as previously described [4]. Informed consent was given by the individual.

## Results

**Fig. 1:**
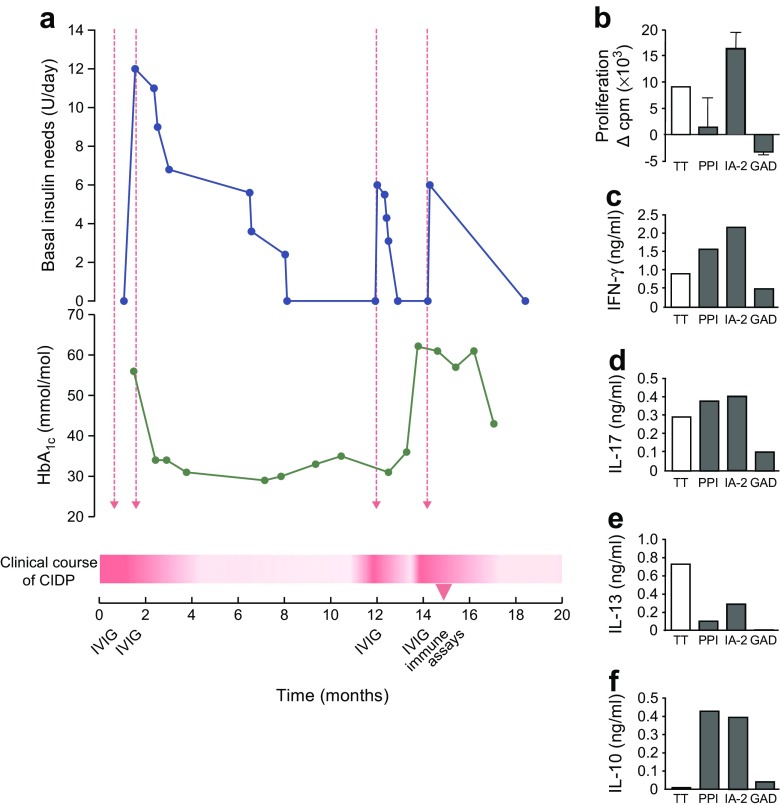
Time course of CIDP disease progression, immunotherapy and type 1 diabetes as defined by insulin need and HbA_1c_ (**a**). During the patient’s most recent flare-up of CIDP, a blood sample was drawn and analysed for T cell reactivity to the islet autoantigens PPI, IA-2 and GAD65 (GAD), as well as T cell responses to tetanus toxoid (TT) as a control for recall immunity to a vaccine antigen unrelated to type 1 diabetes (**b**–**f**). Proliferative responses to PPI were suppressed despite proinflammatory (IFN-γ, IL-17) and anti-inflammatory cytokine production in response to this islet antigen. T cells responded both to IA-2 by proliferation and cytokines, whereas no T cell responses were detectable against GAD65 despite the presence of serum autoantibodies against this protein (not shown). Dashed red arrows indicate IVIG administration. The timeline starts on September 2014 (*t* = 0 months) and ends on May 2016 (*t* = 20 months). The clinical course of CIDP is depicted as a red graded fill, in which the more intensely red areas are flare-ups and white areas are periods of remission. To convert values for HbA_1c_ in mmol/mol into % units, multiply by 0.0915 and add 2.15. Proliferation was normalised to the response in control wells with culture medium and human serum, i.e. without a diabetes-associated antigen (9.5 ± 1.0 [SD] cpm × 10^3^). Cytokine production in control wells was as follows (in ng/ml): IFN-γ, 0.059; IL-17, 0.003; IL-13, 0.006; IL-10, 0.026

In September 2014 (18 months later) the girl experienced a CIDP relapse. She displayed similar symptoms as previously described. She was re-admitted to hospital and received a second round of IVIG treatment (2 g/kg over 5 days). This time serum glucose was elevated to 24 mmol/l (432 mg/dl), HbA_1c_ was 7.6% (60 mmol/mol) and fasting C-peptide was normal (0.57 nmol/l). Furthermore, she tested positive for islet-specific (GAD, IA-2 and zinc transporter 8 [ZnT8]) as well as thyroid-specific (thyroid peroxidase and thyrotropin receptor) autoantibodies, and ketone bodies were found in urine, prompting the diagnosis of Graves’ disease and type 1 diabetes following the criteria defined by the ADA. Her HLA type is also associated with an increased risk for type 1 diabetes and Graves’ disease (HLA-DR3,11; -DQ2,7). She was started on daily insulin injections that normalised her blood glucose levels. However, within 1 month of IVIG treatment her basal insulin needs dropped. Five months later her insulin needs disappeared altogether, her HbA_1c_ normalised and her serum glucose levels remained within the normal range with no further treatment or dietary restrictions (Fig. 1). Her thyroiditis did not respond to IVIG.

In August 2015, 5 months after the last insulin administration, she was re-admitted to hospital for a third CIDP relapse (Fig. 1). Serum glucose levels were elevated at the time of hospitalisation. Insulin treatment was re-established and she underwent another round of IVIG therapy. Again, her basal insulin needs dropped following completion of IVIG treatment and insulin administration was stopped completely 1 month after hospitalisation.

A blood sample that was collected within 1 month of her most recent round of IVIG therapy was analysed for islet autoantigen responses and the presence of T regulatory cells (Tregs) (Fig. 1). T cell proliferation was only noted in response to IA-2, but cytokine responses were detectable against both IA-2 and PPI. Strikingly, proinflammatory cytokine responses (IFN-γ and IL-17) were matched by anti-inflammatory IL-10 production (Fig. 1). Normal levels of Tregs were found (9.2% CD25^high^ CD127^low^ FOXP3^+^ Tregs). Serum analysis revealed circulating autoantibodies to GAD65 (221 U/ml, range < 4 U/ml), IA-2 (1.34 U/ml, range ≤ 1 U/ml) and ZnT8 (287.5 U/ml, range < 15 U/ml) autoantibodies. Islet cell antibody and insulin autoantibody levels were negative. At the time of this report (February 2017), the individual was normoglycaemic, without insulin need and with stable thyroid function.

## Conclusions

Type 1 diabetes is classically described as a progressive disease with irreversible loss of insulin secretory function due to the destruction of beta cells. The present case provides evidence that the natural history of the loss of beta cell function may be more dynamic than the traditional model assumes. We describe relapsing and remitting clinical symptoms in an individual with type 1 diabetes. The capacity to regain complete metabolic control over the course of 18 months implies that residual beta cell mass is sufficient to restore euglycaemia.

It remains unclear to what extent the loss of beta cell function resulted from the loss of beta cell mass as insulin production was completely restored. The current case teaches that insulin insufficiency is not a direct measure of beta cell destruction or mass. Consequently, insulin need as a measure of impaired beta cell function may underestimate beta cell mass.

Suppressed T cell proliferation and robust IL-10 responses to islet autoantigen point to a capacity of the immune system to counter loss of immune tolerance to islets. These residual immunological tolerance mechanisms may be boosted by immunotherapy, even after clinical diagnosis of type 1 diabetes. We previously demonstrated that a T cell response to islets is dominated by IL-10 in non-diabetic donors with a high genetic risk of developing type 1 diabetes, whereas individuals with type 1 diabetes who respond to islet antigens by producing IL-10 develop their disease later in life than those lacking IL-10 responses [5]. Intriguingly, no T cell responses were detectable to GAD65 despite the presence of autoantibodies to GAD65, underscoring a previously noted inverse correlation between T and B cell responses to islet autoantigens [6].

While IVIG might affect peripheral insulin resistance by reducing inflammation, we favour the possibility that immunoregulation contributed to the relapsing and remitting course of diabetes in this individual. Indeed, T cell proliferation to PPI was low and accompanied by IL-10 production, while the immune response against IA-2 was also accompanied by IL-10 production. IVIG may have contributed to immune regulation of diabetes reversing dysfunction of otherwise viable beta cells and restoring normoglycaemia in addition to affecting CIDP. Unlike type 1 diabetes, pathogenic autoantibodies have been identified in CIDP, which could be neutralised by IVIG [3]. Comorbidity of CIDP and type 1 diabetes has been reported previously [7]. Yet in contrast to our present case, all previously reported instances indicate that type 1 diabetes preceded neurological symptoms. In these cases, IVIG greatly improved the neurophysiological symptoms, but metabolic variables remained unaffected [7]. As type 1 diabetes is a T cell-mediated disease in which the role of the humoral response in pathogenicity is still elusive [8], the effectiveness of IVIG is not self-evident. Indeed, IVIG treatment was evaluated as an immune therapy in type 1 diabetes but deemed unviable [9]. The beneficial effect of IVIG treatment was unexpected but replicated (three times in total; Fig. 1). During one of these remissions within 1 month of IVIG treatment, we were able to obtain evidence of immunoregulation of islet autoimmunity. Several mechanisms by which IVIG could affect the immune system have been identified that include activation of natural Tregs and anti-idiotypic antibodies present in IVIG [10].

Our observation that type 1 diabetes may be a relapsing/remitting disease has significant therapeutic implications [2]. Regulatory cytokine production during disease remittance suggests the presence of an antigen-specific regulatory compartment that can be therapeutically exploited. A relapsing/remitting model also implies a period where enough residual beta cell mass exists to restore beta cell function and glycaemic control. Finally, the notion that insulin insufficiency discords with beta cell mass begs reconsideration of the current use of beta cell function as a measure of efficacy for immune interventions aimed at protecting beta cells from autoimmune destruction.

## Abbreviations

CIDP: Chronic inflammatory demyelinating polyneuropathy
GAD65: Glutamic acid decarboxylase 65
IA-2: Islet antigen-2
IVIG: Intravenous immunoglobulin
PBMC: Peripheral blood mononuclear cell
PPI: Preproinsulin
